# Correspondence

**Published:** 1884-10

**Authors:** A. S. Heath

**Affiliations:** Cor. Secretary; New York


### CORRESPONDENCE.

*To the Editor of the Journal of Comparative Medicine and Surgery:*

*To the Editor of the Journal of Comparative Medicine and Surgery:*

DEAR SIR—I trust that you will correct an error which seems to have escaped your usual vigilance in the July number of the JOURNAL. Inclosed I send a copy of the resolutions unanimously passed by the faculty of Columbia College of Comparative Medicine and Surgery that disproves Liautard's announcement that the American Veterinary College had absorbed the Columbia College. I also inclose the Announcement of Columbia College for 1884–85. I have no doubt that you will do Columbia College the justice so respectfully requested.

Yours,

A. S. HEATH, M.D., D.V.S., Cor. Secretary.

New York, Sept. 3, 1884.

